# Digital Hostility Toward LGBTQIA+ Research Recruitment on Social Media Using Topic Modeling and Sentiment Analysis of Facebook Comments: Quantitative Content Analysis Study

**DOI:** 10.2196/79080

**Published:** 2025-09-08

**Authors:** Violeta J Rodriguez, Brett Peterson, Ashley Benhayoun, Qimin Liu

**Affiliations:** 1 Clinical Community Division Department of Psychology University of Illinois Urbana-Champaign Champaign, IL United States; 2 Department of Psychology University of Illinois Urbana-Champaign Champaign, IL United States; 3 Department of Psychological and Brain Sciences Boston University Boston United States

**Keywords:** LGBTQIA+ research, virtual stigma, sentiment analysis, topic modeling, text mining, researcher well-being, stigma communication, Facebook comments, LIWC, Linguistic Inquiry and Word Count

## Abstract

**Background:**

Lesbian, gay, bisexual, transgender, queer/questioning, intersex, asexual (LGBTQIA+) researchers and participants frequently encounter hostility in virtual environments, particularly on social media platforms where public commentary on research advertisements can foster stigmatization. Despite a growing body of work on researcher virtual hostility, little empirical research has examined the actual content and emotional tone of public responses to LGBTQIA+-focused research recruitment.

**Objective:**

This study aimed to analyze the thematic patterns and sentiment of social media comments directed at LGBTQIA+ research recruitment advertisements, in order to better understand how virtual stigma is communicated and how it may impact both researchers and potential participants.

**Methods:**

A total of 994 publicly visible Facebook comments posted in response to LGBTQIA+ recruitment advertisements (January to April 2024) were collected and analyzed. Text preprocessing included tokenization, stop-word removal, and lemmatization. Latent Dirichlet allocation was used to identify latent themes across the dataset. Sentiment analysis was conducted using the Bing Liu and National Research Council lexicons, with scores ranging from –1 (most negative) to 1 (most positive). Linguistic Inquiry and Word Count was used to quantify psychological and moral language features. Comments were also manually coded into four audience target groups (researchers, LGBTQIA+ community, general public, and other commenters), and language category differences were analyzed using 1-way ANOVAs with Bonferroni corrections.

**Results:**

Topic modeling identified three key themes: (1) “Transitions, Health, and Gender Dysphoria,” (2) “Polarized Debate and Response,” and (3) “Religious and Ideological Debates.” Topic 2 had the highest average prevalence (average γ=0.486, SD 0.21). Sentiment analysis revealed negative mean sentiment scores for all three topics: Topic 1 (–0.41, SD 0.48), Topic 2 (–0.21, SD 0.44), and Topic 3 (–0.35, SD 0.46). No topic exhibited a statistically significant predominance of positive sentiment. A 1-way ANOVA showed significant differences in linguistic tone across target groups: negative tone (*F*_3,990_=12.84; *P*<.001), swearing (*F*_3,990_=16.07; *P*<.001), and anger-related language (*F*_3,990_=9.45; *P*<.001), with the highest levels found in comments directed at researchers. Comments targeting LGBTQIA+ individuals showed higher references to mental illness, morality, and threats to children. While affirming responses were less frequent and typically appeared within confrontational contexts, their presence highlights significant moments of solidarity and resistance.

**Conclusions:**

This study documents a persistently hostile virtual environment for LGBTQIA+ research, where researchers are frequently dehumanized and LGBTQIA+ identities are pathologized. These findings reinforce stigma communication models and suggest a need for institutional responses that include mental health support, enhanced moderation tools, and policy advocacy. Future research should investigate how hostile discourse affects researchers’ well-being and recruitment outcomes, and evaluate interventions to foster more respectful engagement with LGBTQIA+ studies.

## Introduction

Over the past 30 years, there has been growing public and scientific recognition of, and interest in, the lives, experiences, challenges, and issues faced by lesbian, gay, bisexual, transgender, queer/questioning, intersex, asexual (LGBTQIA+) individuals. This increased scientific interest has brought greater visibility to LGBTQIA+ communities and scientists along with their research. However, this heightened visibility, coupled with escalating legislative attacks on LGBTQIA+ people across the globe [1], has also intensified the scrutiny and stigma experienced by the broader LGBTQIA+ population, as well as scientists working specifically with these communities [2-4]. Social media platforms have further facilitated the propagation of homotransphobic stigma and discrimination, as demonstrated by the growing literature on virtual hostility against LGBTQIA+ populations [5,6]. While comments sections on social media platforms, such as Facebook, are often defined by high degrees of negative sentiment [7], this vitriol and negativity are pronounced on posts related to LGBTQIA+ topics or identities, where comments regularly reflect themes of suspicion, ridicule, and threats of physical violence toward LGBTQIA+ individuals [8,9]. Therefore, even though increased social media use is linked to a generalized decrease in social and institutional trust [10], these conspiratorial attitudes and antagonistic behaviors are often particularly targeted at, and especially harmful toward, marginalized groups like the LGBTQIA+ community.

Social media has also facilitated the harassment of scientists [11], which has worsened since the COVID-19 pandemic [12]. Scientists researching socially stigmatized groups, particularly those targeted by far-right movements, frequently experience various forms of virtual hostility, including virtual abuse (such as doxing), physical threats, and professional harm [13,14]. These circumstances are especially dire for minoritized, marginalized, and underrepresented scholars, who not only confront threats against their personal identities but also often have limited access to institutional support and resources [4,15,16]. This virtual hostility is further compounded by the intense professional pressures in academia, such as the demands to publish, secure grants, and maintain productivity. The tenure and promotion processes typically fail to account for the barriers created by virtual hostility, whether practical (eg, difficulties with recruitment) or emotional (eg, recovering from the trauma of virtual hostility), leaving these scholars in especially vulnerable positions.

Despite this growing literature on the virtual hostility experienced by researchers generally, less is known about the virtual hostility against researchers who specifically focus on LGBTQIA+ populations. It is known, though, that LGBTQIA+ individuals are underrepresented in science [17] and are more likely to want to leave research-oriented science, technology, engineering, and mathematics fields [18,19]. These LGBTQIA+ researchers also often experience greater isolation, tokenism, and potential cultural barriers to mentoring, funding, or institutional support [20,21]. Therefore, LGBTQIA+ scientists have called for greater support from the scientific community [2].

Three psychosocial frameworks are illustrative for understanding these intergroup dynamics involved in virtual LGBTQIA+ research: Social Identity Theory, the Model of Stigma Communication (MSC), and Stigma Management Communication Theory (SMC). First, Social Identity Theory posits that humans have developed a tendency for group-based thinking, characterized by clear insider-outsider distinctions, to satisfy psychosocial needs related to personal identity and social cohesion [22,23]. Such exclusionary social identities enable in-group members to self-categorization, alleviate existential uncertainty, and balance individual distinctiveness with social inclusion [22]. These identities are formed through “subjective belief systems” and maintained through a series of social sanctions that indicate which actions or characteristics are disqualifying for full group membership [22,24]. Second, the MSC theorizes that the collective expression of stigma plays a fundamental role in the maintenance of these social identities and intergroup dynamics [23,25]. According to proponents of MSC, stigmatization is a method of categorizing a different individual or group as separate from the legitimate social order and thus justifying a negative social posture toward this difference [26,27]. Such stigma can relate to perceived physical, social, or moral differences, and stigmatizers frequently use the visceral emotions of disgust, fear, and anger to virally communicate and enforce this social categorization across in-group social networks [23,27].

Finally, SMC provides a framework for understanding the different methods and approaches stigmatized groups use when responding to such negative social categorization. Since social isolation or rejection often leads to negative physical and mental health outcomes, the SMC framework holds that stigmatized individuals will use similar coping strategies to those encountering other forms of psychological distress, such as internalizing the stigma, avoiding opportunities for stigmatization, or denying the validity of the stigma [27]. These stigma management strategies range widely in their degree of acceptance of the stigma, identification with the stigmatized groups, and sense of personal agency, all factors that shape potential responses and mental health outcomes [25,27]. Therefore, together, these three theories of social identity and intergroup dynamics provide a working framework for understanding why stigmatized identities are developed, how stigmatization is communicated to minoritized groups such as the LGBTQIA+ community, and how stigmatized individuals respond, given the psychological demands of such negative categorization.

These three theoretical steps in the process of stigmatization are evident in the reported reactions and responses to the LGBTQIA+ research virtual. Throughout this paper, we use the term virtual hostility as an umbrella term to describe a broad range of negative or antagonistic virtual interactions directed toward LGBTQIA+ researchers, participants, or research-related advertisements. This includes behaviors such as stigmatizing or hateful comments, targeted harassment, adversarial exchanges, and other forms of interaction that contribute to a hostile virtual environment. Virtual hostility also encompasses comments that vary in tone and emotional intensity; however, it is defined by the function of that negativity—specifically, whether it targets or marginalizes a person or group—rather than by the mere presence of negative language. For example, a comment expressing negative emotion (eg, “I am extremely sad”) or tone (eg, “I hate that this happened”) would not qualify as hostile unless it actively targets others (eg, “I hate that person” or “I am extremely sad about what those people have done to our country”). Accordingly, we use virtual hostility to refer to actions that target or marginalize others in virtual spaces, and we use negativity to describe the emotional tone, content, or valence of a comment more generally.

The virtual hostility directed toward researchers and LGBTQIA+ individuals clearly falls within the outlined parameters of stigmatization in which posters signal their social identity by attacking these groups as outside the proper social order and then using outrage or disgust to virally spread this sentiment among their virtual social network. Thus, these social media forums provide a unique venue for examining responses to virtual stigmatization, since they expose researchers and potential participants directly to stigma while attempting to participate in research efforts aimed at addressing or alleviating it. According to the theorized SMC framework, this dynamic may deter potential participants from engaging in such research or discourage researchers from pursuing it further to avoid personal stigmatization and its psychological impact. Alternatively, LGBTQIA+ individuals or allies may also directly respond to such attacks in order to signal their social identity or deny the stigmatization, which could have its own effects on the intended research projects. Therefore, analyzing virtual reactions to LGBTQIA+ research offers an opportunity to validate and expand existing frameworks regarding the communication of stigma and corresponding responses within an underresearched medium and population.

Our research laboratories recently received a large number of hostile comments from people responding to our social media page advertisements aimed at recruiting LGBTQIA+ participants for studies, providing a tangible example of the current state of virtual research with LGBTQIA+ communities. The negativity expressed in these comments not only highlights the prevalence of virtual hostility but also offers an opportunity to examine its effects on research recruitment efforts and the participation of LGBTQIA+ individuals in scientific studies. Understanding the nature of these hostile comments is thus crucial, as these virtual dynamics not only affect the well-being and productivity of researchers but also reflect the broader environment facing LGBTQIA+ communities.

Therefore, by analyzing the content of these comments, we aim to provide insights into the nature of virtual stigma and discrimination perpetrated by individuals against LGBTQIA+ people, offering a unique lens through which to view and understand discrimination. Although the literature has documented incidents of virtual hostility related to LGBTQIA+ research [4], no studies to our knowledge have examined the content of such hostile responses to LGBTQIA+ research. This gap is particularly relevant to this study’s objectives, as understanding these hostile dynamics can inform strategies to mitigate the barriers to LGBTQIA+ research participation and improve engagement with stigmatized groups. Therefore, analyzing the content and degree of negativity in such comments is vital for understanding how such reactions impact researchers and potential participants, as past research has demonstrated that the presence of hostile comments can influence how people view and engage with posts [28,29].

Given this, here we use topic modeling and sentiment analysis to examine the comments directed at LGBTQIA+ researchers and social media recruitment advertisements intended to recruit LGBTQIA+ parents and their children. Topic modeling is a statistical technique that helps uncover the underlying themes or topics within a large corpus of text, and sentiment analysis is a technique that then classifies the expressed opinions as positive, negative, or neutral. By integrating these methods, we aim to provide an understanding of the thematic content and emotional tone of the comments received regarding LGBTQIA+ research.

## Methods

### Study Design

#### Recruitment

We extracted comments from our recruitment advertisements on social media to recruit LGBTQIA+ participants for parent-child studies. A total of five advertisements were part of a coordinated effort to recruit LGBTQIA+ participants for a series of parent-child studies focused on family dynamics and child development. Because all advertisements targeted the same population and research topic, the comments reflect public responses to a consistent line of inquiry. Thus, the text data involved Facebook comments on recruitment advertisements from January 2024 to April 2024, which accumulated to a total of 994 comments during this period. While our research team had similar recruitment advertisements posted on other social media platforms, such as Instagram, Facebook was the only platform which does not allow users to disable comment sections on such research recruitment advertisements. Comments on Instagram, on the other hand, were disabled by our team. Additionally, there is a robust literature exploring the negativity of Facebook comment sections and how this sentiment shapes user engagement [7,9,28]. Therefore, we choose to focus this research project specifically on Facebook comments, because it both aligned with our data and situated our findings within the broader literature on negativity in Facebook comments.

#### Manual Coding Process

To contextualize the automated Linguistic Inquiry and Word Count (LIWC) findings and facilitate comparative analyses across different target audiences (eg, researchers, LGBTQIA+ community, and general public), we conducted a manual coding process to identify the primary audience or target of each comment. This coding was carried out by two trained team members: a PhD student in clinical psychology and a laboratory manager with a master’s degree in social psychology. Both coders independently reviewed an initial set of 150 comments to establish a shared understanding of the coding criteria and reached high interrater reliability (Cohen κ=0.85). After establishing reliability, the remaining comments were divided and coded independently. This process enabled us to systematically assign each comment to one of four audience groups (researchers, LGBTQIA+ Community, other commenters, and general public), which in turn allowed for meaningful group comparisons using the LIWC output. By combining human judgment with automated linguistic analysis, we were able to more precisely capture the emotional tone and thematic content of comments directed at distinct groups.

### Ethical Considerations

The data used in this study were collected from the first author’s laboratory’s publicly available social media pages, and no personally identifiable information was included in the analysis. Given that we used publicly accessible, deidentified comments from Facebook without engaging with the participants in any form, the institutional review board did not deem it as requiring additional approval. Nonetheless, we carefully present the analyses and results in this paper, avoiding any personally identifiable information and paraphrasing quotes to maintain anonymity.

### Positionality Statement

The results presented in this paper are solely intended to support our findings. However, some content may be sensitive, and we advise readers to approach it with caution. Nevertheless, it is due to its sensitive nature that we chose to present these findings, as our goal is to bring awareness to the experiences of researchers working with LGBTQIA+ communities. In addition, the results presented in this paper are solely intended to support our findings. However, we acknowledge that some content may be sensitive in nature, particularly for those who have lived experience within or conduct research on LGBTQIA+ communities. We encourage readers to approach the material with care and reflection. It is precisely because of this sensitivity that we chose to share these findings—to bring attention to the often-overlooked experiences of researchers working with and within LGBTQIA+ communities.

Our research team is composed predominantly of people of color, immigrants, and sexual and gender minority individuals. Many of us hold multiple marginalized identities. These lived experiences shape our research priorities, inform our interpretation of data, and ground our work in a deep commitment to equity, representation, and ethical engagement. Several authors identify as LGBTQIA+, which allowed us to approach this study with heightened cultural humility and an awareness of the concerns facing LGBTQIA+ communities and scholars. Several coders and analysts on our team identify as members of the LGBTQIA+ community, which informed both our approach to data engagement and our interpretation of sensitive material in this project. The overwhelmingly hostile responses to the recruitment advertisements were largely unanticipated, making it difficult to fully shield LGBTQIA+ researchers from emotional strain during the initial wave of negative comments. However, this experience ultimately galvanized our team’s commitment to the project and led to a more intentional, trauma-informed approach to data analysis. To support researchers’ well-being and minimize potential bias, we adopted a collaborative coding process. Hostile comments were reviewed and analyzed in pairs, enabling researchers to share the emotional burden of content that directly targeted their identities, process it at a manageable pace, and debrief together when needed. This paired approach reduced individual strain while fostering mutual support. Additionally, integrating multiple human coders with topic modeling and sentiment analysis tools further mitigated the influence of personal bias and enhanced the rigor of our interpretation.

### Data Preprocessing

Before analysis, the collected text data underwent preprocessing steps customary to text analyses to ensure quality and consistency, including cleaning for nontextual elements (eg, emojis and images), lowercasing, tokenization, stop-word removal, stemming, and lemmatization [30].

### Analysis

To uncover the underlying themes in the comments, we used the latent Dirichlet allocation (LDA) model. To determine the optimal number of topics, we systematically tested a range of topic counts (2 to 10, 15, and 20) and evaluated model performance using four established metrics: Griffiths2004, CaoJuan2009, Arun2010, and Deveaud2014 (via the ldatuning package). CaoJuan2009 and Arun2010 prioritize minimizing topic density and redundancy, while Griffiths2004 and Deveaud2014 assess model likelihood and semantic coherence, respectively [31,32]. The final model was selected based on the convergence of multiple metrics (lowest score for CaoJuan2009, highest for Deveaud2014) and human interpretability. We reviewed the top terms and coherence of topic themes across models with 3 to 6 topics. We ultimately selected the 3-topic solution for its optimal balance between statistical fit and interpretability. To assess thematic robustness, we conducted a sensitivity analysis by comparing models with 2 to 6 topics and reviewing the stability of major themes across these solutions. Themes identified in the 3-topic model remained substantively similar in neighboring models (eg, in the 4-topic model, one theme split into subclusters, but the core concepts remained intact), suggesting thematic stability. To interpret the thematic content, the top 10 terms for each topic were extracted. These terms, along with representative comments, were reviewed collaboratively by our research team to assign topic labels based on recurring linguistic patterns and comment tone. The average proportion (γ) of each topic from the LDA model that comprises each comment was also calculated to determine the prominence of each topic.

Next, sentiment analysis was conducted to classify the expressed opinions in the comments as positive, negative, or neutral. We used both the Bing Liu Sentiment Lexicon and the National Research Council Word-Emotion Association Lexicon to conduct such an analysis [33,34]. These are two well-established sentiment lexicons, which include large wordpools (6800 and 14,000, respectively) and are widely used in text mining projects [33,35]. Each tokenized word was matched with the sentiment lexicon to assign a sentiment score. The overall sentiment for each comment was then calculated by summing the sentiment scores of its constituent words. These sentiment scores range from –1 (negative) to 1 (positive). Additionally, we computed sentiment scores of each topic by averaging sentiments from comments with a predominant topic. To further understand the context of the comments by topic, we performed an n-gram analysis focusing on the most prominent bigrams (two-word phrases) per topic. This analysis provided insights into commonly occurring word pairs, highlighting specific themes and sentiments that may not be evident from individual words alone.

To supplement sentiment and topic modeling, we also used LIWC, a widely used natural language processing tool for systematic, data-driven text analysis [36]. LIWC provides consistent, reproducible, and theory-driven insights by quantifying the frequency of words in psychologically meaningful categories (eg, sentiment, anger, swearing, moral reasoning, and political discourse). Compared to manual coding, LIWC reduces human bias, enhances replicability, and allows for the efficient processing of large-scale datasets. Given the nature of the dataset—public comments on social media regarding sexual and gender minority research—LIWC was particularly advantageous in detecting patterns in sentiment, conflict, and hate speech markers while minimizing individual coder bias. The categories selected (eg, tone_neg, emo_anger, swear, moral, and conflict) align with prior research on virtual hostility and discrimination [37]. Moreover, using LIWC allowed us to directly compare linguistic trends in supportive versus hostile responses, an analysis that was difficult to standardize with manual coding. We then used 1-way ANOVAs to assess whether LIWC-derived language categories differed significantly across manually coded audience groups with Bonferroni corrections for multiple comparisons.

All data analysis and visualization were conducted using R (R Core Team), with packages including topicmodels [38], tidytext [39], ggplot2 [40], and dplyr [41].

## Results

### Topic Modeling With LDA

Evaluation metrics on the LDA models showed that 3 topics emerged as the optimal number of topics, which offered the best trade-off between simplicity and topic interpretability. The top 10 terms for each topic identified by the LDA model are presented in Table 1. Table 1 also shows that Topic 2 was the dominant topic with an average γ value of 0.486 (SD 0.21). To illustrate the nature of the comments analyzed and how the topics were labeled, we include a few representative (and paraphrased) quotes from the dataset.

**Table 1:** Thematic analysis of Facebook comments on LGBTQIA+^a^ research recruitment advertisements using latent Dirichlet allocation (United States, January to April 2024)^b^.

Topic	Label	Average γ (SD)	Top terms
1	Transitions, Health, and Gender Dysphoria	0.272 (0.19)	kids, people, child, mental, transition, gender, understand, think, puberty, transitioning
2	Polarized Debate and Response	0.486 (0.21)	clown, ignorant, keep, people, see, right, said, sex, little, love
3	Religious and Ideological Debates	0.242 (0.18)	people, god, kids, trans, women, men, community, everyone, gender, think

^a^LGBTQIA+: lesbian, gay, bisexual, transgender, queer/questioning, intersex, asexual.

^b^This table presents the results of a topic modeling analysis conducted on 994 publicly visible Facebook comments posted in response to LGBTQIA+-focused research recruitment advertisements shared by a US-based academic research team between January and April 2024. LDA was used to identify three dominant thematic clusters within the comments. Each topic is labeled based on its most salient linguistic content, with the 10 most frequent terms listed to illustrate key language features. The average γ column indicates the average γ score (ie, the proportion of each topic present) across all comments, reflecting the relative prominence of each theme in the dataset. Topic 1 (“Transitions, Health, and Gender Dysphoria”) included stigmatizing references to mental health and youth transitions; Topic 2 (“Polarized Debate and Response”) captured adversarial, mocking, or inflammatory language used in direct exchanges; and Topic 3 (“Religious and Ideological Debates”) centered on cultural and political discourse related to gender and sexuality. This analysis highlights the nature and emotional tone of virtual discourse encountered by researchers and LGBTQIA+ communities during participant recruitment efforts.

First, Topic 1, labeled “Transitions, Health, and Gender Dysphoria,” included comments stigmatizing or pathologizing LGBTQIA+ people, such as “This is the most significant societal delusion we’ve experienced since the era of witch hunts,” “LGBTQIA+ parents simply don’t exist,” and “Leave the children alone. You deviants are not going to make this stuff seem normal.” These Topic 1 responses largely focused on directly attacking or criticizing either the researchers or the whole LGBTQIA+ community. The hostile comments toward researchers ranged from general suspicions about why they were allowed to advertise on Facebook, such as “Why does Facebook allow so many ads from child groomers?” to more direct accusations or threats, for example, “Throw these people in jail.” Then, the Topic 1 comments attacking the broader LGBTQIA+ community were often even more vitriolic, labeling it as full of “deviants” or “child groomers” and then invoking threats of physical harm.

Topic 2, labeled “Polarized Debate and Response,” included adversarial comments or exchanged insults, such as “[Name], you aren’t actually gay; you’re a predator,” and “Laughing out loud! The concept of LGBTQIA+ parents is absurd,” and “Learn the facts about transitioning before you make yourself look more ignorant and clueless.” These Topic 2 comments largely focused on criticizing or responding to other commenters to defend LGBTQIA+ people, rather than being directed toward the researchers or broader community as in Topic 1. Many of these responses came from anti-LGBTQIA+ commenters attacking those supportive of these communities, such as “You won’t listen to anyone else’s perspective. It’s crazy to let kids that age make such big decisions about their health.” However, other Topic 2 comments came from LGBTQIA+ allies defending the rights of these communities or attacking the beliefs of those anti-LGBTQIA+ commenters. Some of these allies’ comments focused on positive affirmations or genuine explanations of LGBTQIA+ experiences, such as “I’m open to discussing this and clearing things up” and “Gender is a social concept that’s only loosely tied to biological sex.” Many other comments from allies, however, were still quite negative, using epithets such as “sky daddy” and “wannabe pharisee” to criticize their opponents’ religious beliefs.

Finally, Topic 3, labeled “Religious and Ideological Debates,” often included comments connected to wider cultural debates, such as, “It’s difficult to argue you’re being harassed when you’re initiating these discussions,” “Stop responding and allow people to live their own lives,” and “Democrats want ... sports ruined by transgenders, bathroom stalkers, more drugs, more abortions, endless spending...” These Topic 3 comments mostly focused on engaging with some larger social, political, or ideological conflict, rather than attacking those specific researchers or commenters. In many cases, these comments simply asserted some firmly held religious or ideological belief related to gender, society, or god, such as “God created only two genders, male and female.” Many of the issues invoked in these comments, however, were either only tangentially related to the topics of the research, such as attacking drag queens or trans people in sports, or seemingly not related to the research at all, such as public spending in the US context or the evangelical pathway to salvation.

### Sentiment Analysis

The sentiment analysis of comments by predominant topics revealed uniformly negative sentiment scores across all topics. Across the 994 publicly visible Facebook comments analyzed, Topic 1 (“Transitions, Health, and Gender Dysphoria”) had the most negative mean sentiment score (–0.41, SD 0.48), followed by Topic 3 (“Religious and Ideological Debates”) at –0.35 (SD 0.46). Topic 2 (“Polarized Debate and Response”) had the least negative mean score (–0.21, SD 0.44). These values indicate that, although the intensity of negativity varied by topic, all discussions leaned toward a negative emotional tone. Comments were assigned to their dominant topic based on the highest γ value from LDA topic modeling, and sentiment was calculated using lexicon-based sentiment analysis (Bing Liu and National Research Council Lexicons), with scores ranging from –1 (most negative) to 1 (most positive). This pattern underscores the predominance of negativity in public social media discourse surrounding LGBTQIA+ research recruitment between January and April 2024.

**Figure 1:**
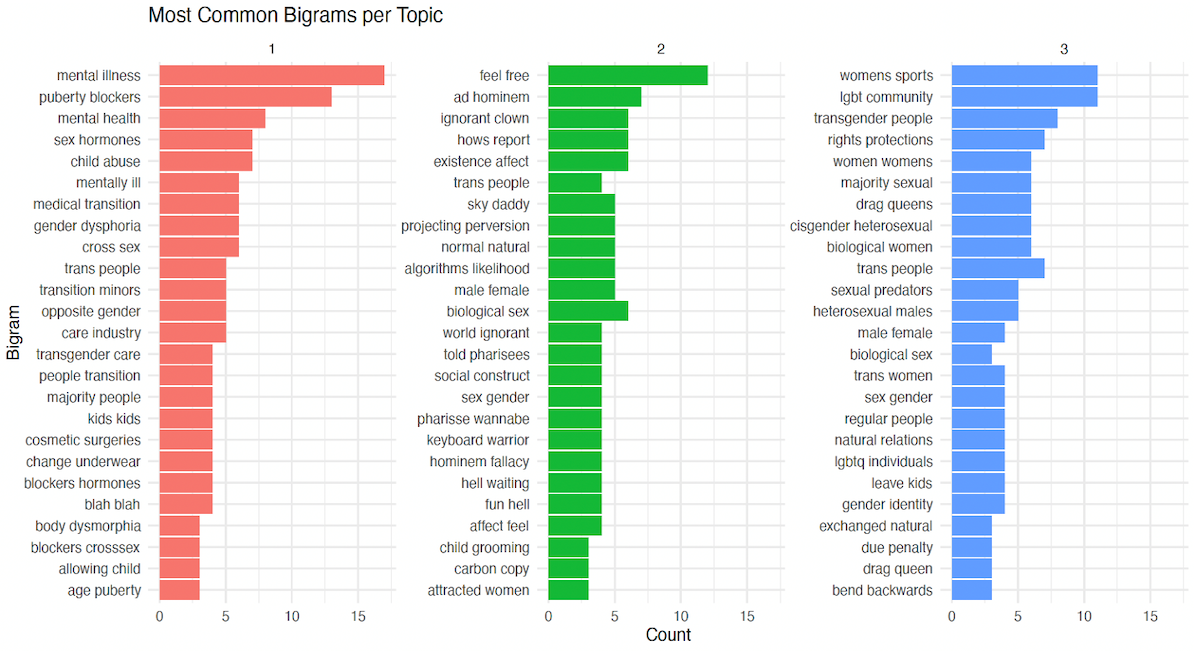
Bigrams in Facebook comments responding to LGBTQIA+ research recruitment advertisements (United States, January to April 2024). This figure displays the most frequently occurring bigrams (two-word combinations) in 994 publicly visible Facebook comments posted in response to LGBTQIA+-focused research recruitment advertisements by a US-based academic research team between January and April 2024. Comments were analyzed using latent Dirichlet allocation topic modeling, and each comment was assigned a dominant topic based on the highest gamma value. The bars represent the frequency of each bigram within its respective topic. In Topic 1 (“Transitions, Health, and Gender Dysphoria”), bigrams such as “mental illness,” “puberty blockers,” and “child abuse” reflect pathologizing and stigmatizing language. Topic 2 (“Polarized Debate and Response”) includes bigrams like “ad hominem,” “ignorant clown,” and “sky daddy,” which illustrate mocking or adversarial discourse, often in exchanges between commenters. Topic 3 (“Religious and Ideological Debates”) features bigrams such as “women’s sports,” “lgbt community,” and “rights protections,” highlighting the connection between LGBTQIA+ research and broader ideological or political debates. This figure demonstrates how specific language patterns are associated with distinct types of stigma communication in public discourse on LGBTQIA+ research. Bar colors correspond to the dominant topic: red (Topic 1), green (Topic 2), and blue (Topic 3). LGBTQIA+: lesbian, gay, bisexual, transgender, queer/questioning, intersex, asexual.

To further understand the context and sentiment of the comments directed at LGBTQIA+ researchers and recruitment advertisements, we performed an n-gram analysis focusing on the most common bigrams (two-word phrases) per topic. This analysis helped identify commonly occurring word pairs, providing greater insights into the themes and sentiments expressed. Figure 1 presents the most common bigrams for each identified topic. For Topic 1, frequent bigrams include “mental illness,” “puberty blockers,” “mental health,” and “sex hormones,” with “mental illness” appearing most often. In Topic 2, prominent bigrams are “feel free,” “ad hominem,” “ignorant clown,” and “hows report” (related to the intention to report the advertisements), with “feel free” being the most frequent. Topic 3 features bigrams like “women’s sports,” “drag queens,” “cisgender heterosexual,” and “rights protections,” with “women’s sports” and “LGBT community” as the most common. Finally, to further quantify the virtual hostility into meaningful categories, we used LIWC (Table 2). LIWC findings suggest that virtual hostility directed at researchers and the LGBTQIA+ community tended to be more intense and negative, specifically dehumanizing, vulgar, and informal. Additionally, this virtual hostility tended to use netspeak and focused on topics such as mental illness and morality.

This table presents mean scores and SDs of LIWC variables derived from 994 publicly visible Facebook comments posted in response to LGBTQIA+-focused research recruitment advertisements disseminated by a US-based academic team between January and April 2024. Comments were manually coded into four primary target audiences—researchers, LGBTQIA+ community members, other commenters, and the general public—and analyzed for linguistic content using LIWC. Variables span sentiment (eg, positive or negative tone), emotion (eg, anger and sadness), social or moral reasoning, swearing, references to family, illness, politics, and gender. The final column includes *F*-statistics and Bonferroni-corrected *P* values from 1-way ANOVAs comparing audience groups. Superscripts denote significant post hoc differences between specific audience pairs. This analysis highlights how the tone and content of virtual stigma communication vary by target group, with especially high levels of negative affect, swearing, and moral language in comments directed at researchers and LGBTQIA+ individuals.

**Table 2:** Linguistic patterns across Facebook comments directed at different audience groups in response to LGBTQIA+^a^ research recruitment advertisements (United States, January to April 2024).

Category	Researchers, mean (SD)	LGBTQIA+ community, mean (SD)	Other commenters, mean (SD)	General public, mean (SD)	*F* test (*df*)	*P* value
Threading Depth	2 (—^b^)	1.83 (0.41)	1.83 (0.38)	—	0.10 (2, 797)	.90
Segment	1 (0)	1 (0)	1 (0)	1 (0)	— (3, 984)	—
Power^c,d,e^	1.35 (3.64)	2.14 (5.05)	1.27 (3.23)	5.89 (12.87)	8.42 (3, 941)	<.001
Cause^f^	1.08 (3.01)	0.58 (2.17)	1.61 (2.87)	1.89 (3.39)	3.28 (3, 941)	.02
Discrep^g^	3.16 (7.09)	2.48 (5.75)	1.63 (3.19)	1.58 (2.92)	3.89 (3, 941)	.009
Tentat^f^	1.01 (3.43)	0.54 (2.21)	1.72 (2.84)	0.42 (1.15)	5.30 (3, 941)	.001
Affect^g,h^	15.95 (21.32)	11.10 (16.53)	6.26 (6.95)	9.31 (14.08)	23.20 (3, 941)	<.001
Tone—Positive	3.27 (8.14)	2.67 (5.84)	2.65 (4.64)	3.02 (6.58)	0.30 (3, 941)	.82
Tone—Negative^f,g^	7.97 (15.96)	6.26 (15.25)	2.92 (4.75)	6.29 (13.33)	12.93 (3, 941)	<.001
Emotion^g,h^	4.01 (9.96)	1.22 (3.73)	2.16 (4.31)	2.90 (6.62)	3.77 (3, 941)	.01
Emotion—Positive^c,d^	0.23 (1.05)	0.31 (1.54)	0.91 (2.48)	2.27 (6.71)	4.14 (3, 941)	.006
Emotion—Negative^g,h^	3.23 (9.21)	0.88 (3.47)	1.12 (3.44)	0.63 (1.37)	5.40 (3, 941)	.001
Emo_anx	0.51 (3.30)	0.02 (0.13)	0.24 (2.37)	0 (0)	0.53 (3, 941)	.66
Emotion—Anger	1.02 (6.54)	0.03 (0.14)	0.40 (1.61)	0 (0)	2.34 (3, 941)	.07
Emotion—Sad	0.15 (1.17)	0.43 (2.65)	0.18 (1.21)	0.48 (1.30)	0.92 (3, 941)	.43
Swear^c,g,h^	5.30 (15.46)	2.14 (8.18)	0.63 (2.26)	0 (0)	18.32 (3, 941)	<.001
Socbehav^c,g^	7.71 (15.60)	4.41 (8.74)	5.06 (6.15)	1.96 (4.17)	3.69 (3, 941)	.01
Prosocial^c,g,h^	3.07 (9.28)	1.18 (4.94)	0.64 (2.06)	0.41 (1.18)	10.60 (3, 941)	<.001
Polite	1.11 (6.03)	0.15 (1.12)	0.38 (2.45)	0 (0)	1.66 (3, 941)	.17
Conflict	0.10 (0.59)	0.10 (0.52)	0.45 (1.75)	0.19 (0.74)	1.77 (3, 941)	.15
Moral^g^	3.01 (13.23)	2.08 (7.03)	1.11 (2.82)	0.29 (1.12)	4.09 (3, 941)	.007
Comm^f,g^	0.64 (4.32)	0.09 (0.41)	1.97 (3.40)	1.07 (3.69)	8.95 (3, 941)	<.001
Family^g,h^	0.88 (2.91)	2.38 (7.29)	0.39 (1.95)	0.61 (2.35)	10.96 (3, 941)	<.001
Female	1.67 (12.91)	0.34 (1.28)	0.46 (1.97)	0.15 (0.59)	2.06 (3, 941)	.10
Male^c,d,e^	0.07 (0.56)	0.43 (1.48)	0.97 (3.02)	3.48 (12.88)	5.13 (3, 941)	.002
Culture	1.81 (12.94)	0.55 (4.10)	0.78 (3.05)	1.03 (2.51)	1.10 (3, 941)	.35
Politic	0.14 (1.08)	0.54 (4.10)	0.25 (1.23)	1.03 (2.51)	1.89 (3, 941)	.13
Ethnicity^g^	1.67 (12.91)	0 (0)	0.22 (2.01)	0 (0)	3.00 (3, 941)	.03
Tech	0 (0)	0.01 (0.05)	0.31 (1.97)	0 (0)	1.14 (3, 941)	.33
Religion	1.23 (5.74)	2.21 (6.61)	0.97 (3.82)	1.41 (3.97)	1.83 (3, 941)	.14
Illness^f^	1.16 (5.20)	1.69 (12.34)	0.28 (1.82)	0.35 (1.36)	3.41 (3, 941)	.02
Mental^f,g^	3.18 (10.75)	4.33 (12.74)	0.43 (2.47)	1.16 (3.13)	17.75 (3, 941)	<.001
Sexual	0.79 (2.80)	2.26 (9.11)	0.95 (3.91)	2.82 (4.96)	2.64 (3, 941)	.05
Death	0.07 (0.54)	0.14 (0.92)	0.07 (0.46)	0.19 (0.74)	0.61 (3, 941)	.61
Risk	0.24 (1.84)	0.10 (0.59)	0.21 (1.06)	0.12 (0.46)	0.25 (3, 941)	.87
Focus—Past	1.32 (6.78)	0.85 (2.30)	1.76 (3.91)	1.77 (3.87)	1.18 (3, 941)	.32
Focus—Present^d^	6.49 (10.93)	4.21 (7.01)	6.15 (5.58)	10.25 (13.01)	4.21 (3, 941)	.006
Focus—Future	1.10 (3.48)	0.41 (1.49)	1.09 (3.11)	0.15 (0.59)	1.43 (3, 941)	.23
Netspeak^g,h^	3.28 (13.83)	0.74 (2.98)	0.93 (2.84)	0 (0)	5.72 (3, 941)	<.001
Exclam^f,g^	5.89 (23.22)	10.01 (40.64)	1.04 (5.95)	0.95 (3.69)	11.04 (3, 941)	<.001

^a^LGBTQIA+: lesbian, gay, bisexual, transgender, queer/questioning, intersex, asexual.

^b^No value was calculated.

^c^Difference between the general public and researchers.

^d^Difference between the general public and the LGBTQIA+ community.

^e^Difference between the general public and other commenters.

^f^Difference between the LGBTQIA+ community and other commenters.

^g^Difference between researchers and other commenters.

^h^Difference between researchers and the LGBTQIA+ community.

## Discussion

### Principal Results

The results of this study provide an understanding of the thematic content and sentiment of comments directed at LGBTQIA+ researchers and social media recruitment advertisements recruiting LGBTQIA+ parents. The integration of topic modeling and sentiment analysis revealed significant insights into the nature of these comments, highlighting the challenges and hostility faced by both researchers and the broader LGBTQIA+ community on social media platforms.

We identified three distinct themes within the comments via topic modeling. Topic 1 was characterized by terms related to mental health and transitions, with the bigram highlighting phrases like “mental illness,” “child abuse,” and “sex hormones.” These Topic 1 comments thus reflect how frequently LGBTQIA+ identities are characterized as forms of mental illness or child abuse and suggest a significant presence of misinformation and myths about LGBTQIA+ issues, like hormone treatments and gender dysphoria. The Topic 1 comments also frequently attacked the researchers or LGBTQIA+ people directly, accusing them of nefarious intent and invoking the possibility of violent retribution. This explains why Topic 1 scored as the most negative topic, since most comments were direct, vitriolic attacks on supposed cultural enemies or threats. The dynamics in these comments, therefore, also align with the existing literature on Facebook, which suggests the platform particularly heightens negative reactions to larger, “shadowy” groups even more than specific individuals [42].

Topic 2 was characterized by confrontational and derogatory language, with frequent bigrams such as “ignorant clown,” “ad hominem,” and “projecting perversion,” alongside phrases like “existence affect” that denied the legitimacy of LGBTQIA+ identities. Despite this hostile tone, Topic 2 had the least negative average sentiment score, likely due to the presence of LGBTQIA+ allies who, while often engaged in adversarial exchanges, contributed comments that included both affirming statements (eg, “I’m open to discussing this and clearing things up”) and hostile rebuttals (eg, “sky daddy” and “wannabe pharisee”). These debates—frequently between allies and opponents—generated a mix of sentiments, accounting for the topic’s comparatively lower negativity. The prevalence of this dynamic also explains why Topic 2 emerged as the largest theme, as these exchanges often evolved into extended back-and-forth arguments that amplified engagement. Ultimately, Topic 2 illustrates both the ongoing contestation of LGBTQIA+ legitimacy in public discourse and the tendency for research-related content to trigger polarizing debates that stray far from the original focus of recruitment.

Topic 3 focused on community and rights-related issues, with common terms such as “god,” “community,” and “gender.” The common bigrams, like “rights protections,” “drag queens,” and “women’s sports,” underscore the ongoing debates about LGBTQIA+ rights and inclusion both in virtual and offline spaces. These Topic 3 comments often asserted deeply held religious beliefs or referenced various ideological debates only tangentially related to the research topic. Despite the wide-ranging nature of these topics, however, these different comments similarly reflected an unspoken sense that recognizing LGBTQIA+ identities is a threat to these beliefs and that the very nature of this research inherently positions it as on the opposing side of some political or ideological schism. These comments, therefore, are consistent with the broader discourse seen in the media regarding the use of religious and ideological beliefs opposing LGBTQIA+ identities, which have sparked anti-LGBTQIA+ legislation across the globe. Topic 3 comments also align with the existing research on Facebook, which suggests that the nonanonymous nature of Facebook comments may actually increase negativity when attacking supposed outsiders becomes an indicator of in-group status [42].

In addition to identifying major themes and sentiment, our LIWC analysis provided a more granular understanding of the linguistic patterns used in hostile comments and the differential targeting of researchers versus the LGBTQIA+ community more broadly. Comments referencing researchers exhibited the highest rates of negative affect (eg, Affect, Tone—Negative, Emotion Negative) and contained elevated levels of swearing and vulgarity (Swear; mean 5.30, SD 15.46), compared to those targeting LGBTQIA+ individuals directly or other commenters. This pattern of linguistic intensity reinforces the notion that researchers were frequently dehumanized and framed as moral violators or societal threats—consistent with the MSC, where disgust and moral outrage are mobilized to justify exclusion.

Furthermore, language toward the LGBTQIA+ community showed increased references to family, illness, and morality, suggesting attempts to stigmatize these identities through appeals to normative values and perceived health risks. For example, elevated scores on Family, Mental, and Moral categories indicate that commenters often framed LGBTQIA+ existence as a danger to children or as pathological. This aligns with the Topic 1 content, where phrases such as “mental illness” and “child abuse” were recurrent.

Interestingly, while the general public comments reflected somewhat lower negativity in tone overall, the presence of Netspeak, Exclamations, and Focus on the Present among researchers’ and LGBTQIA+ individuals’ comments suggests the comments were emotionally charged and reactionary. Such findings illustrate how hostile virtual environments not only reproduce social stigma but do so through emotionally inflammatory and morally charged rhetoric—a mechanism known to exacerbate psychological distress and potentially deter continued engagement in public-facing research [43,44].

While it is reasonable to ask whether the intensity or nature of hostility toward LGBTQIA+ research differs meaningfully from other sensitive health topics (eg, mental health, reproductive health, and vaccine research), direct comparisons remain challenging due to the limited number of empirical studies specifically analyzing virtual responses to this research, and instead these focus on general virtual discourse. Our own literature search revealed a relative paucity of studies that systematically examine public comment sentiment, hate speech patterns, or linguistic hostility in response to LGBTQIA+ research recruitment compared to the broader (though still emerging) literature on virtual harassment in other health domains. Future work that applies parallel analytic methods across different stigmatized health domains is needed to more rigorously assess the degree and distinctiveness of virtual hostility directed at LGBTQIA+ research and to contextualize these dynamics within the broader landscape of health communication.

### Limitations

Despite the novel contributions of this study, several limitations must be acknowledged. First, the dataset was restricted to Facebook comments in response to recruitment advertisements posted by a single research laboratory over a 5-month period. As such, the findings may not generalize to other platforms (eg, X or Twitter and TikTok), timeframes, or forms of virtual engagement (eg, private messages and quote posts). Second, although the comments were paraphrased to protect anonymity, their content may still reflect selection bias due to Facebook’s algorithms and the specific audiences reached by the advertisements. Third, our sentiment and topic modeling analyses relied on lexicon-based approaches that, while widely used, may not fully capture the nuanced tone of sarcasm, coded language, or regional vernacular often present in hostile virtual discourse. Fourth, although manual coding allowed us to contextualize linguistic patterns by audience group, assigning a single intended target to each comment may oversimplify the complex social dynamics at play in public comment threads. Finally, the cross-sectional nature of the dataset prevents conclusions about changes over time or the longitudinal impact of virtual hostility on researcher well-being or participant engagement. Future studies using longitudinal and experimental designs could expand on these findings to better understand causal mechanisms and potential interventions.

Importantly, while our analyses focused on the content, sentiment, and linguistic features of visible Facebook comments, we did not capture data on platform-level dynamics such as post reach, algorithmic suppression, or user reporting behaviors. Notably, our manual coding did not reveal any explicit calls for reporting or flagging LGBTQIA+-related research advertisements within the sampled comments. However, we recognize that hostile users may still engage in such actions off-comment or via coordinated reporting, which could trigger algorithmic downgrading or content removal. This possibility has important implications for the dissemination and reach of LGBTQIA+ health research on social media. Future research should investigate whether LGBTQIA+ content is disproportionately subject to moderation or reduced visibility compared to other health topics, and how hostile user engagement may contribute to those outcomes [45-49].

Additionally, although timestamps were available for each comment, our dataset spanned a relatively brief time frame (January-May 2024), and comments were heavily clustered within a few days following each advertisement’s posting. Within the limited timeframe of data collection, our team did not observe any meaningful temporal shifts in the hostility of reactions, largely due to the consistently high levels of negativity expressed across the comment sections on days when the advertisements were posted. As previously noted, these sections often followed a predictable cycle: an initial inflammatory comment would trigger a wave of hostile exchanges, which would subside before being reignited by subsequent provocative posts. However, because negativity permeated every phase of this cycle, it was difficult to determine whether hostility genuinely intensified or diminished over time. Moreover, the narrow temporal scope and limited volume of data constrained our ability to assess fluctuations in comment tone or link them to external sociopolitical events (eg, new legislation, media controversies, or Pride Month). Additionally, the limited temporal range and density precluded meaningful analyses of how comment tone may have fluctuated over time or in response to external sociopolitical events (eg, legislation, media controversies, and Pride Month). Future studies would benefit from extended periods to capture temporal variation in virtual discourse. Similarly, our analyses focused exclusively on textual content and did not incorporate nonverbal or visual elements such as Facebook reaction emojis (eg, “Haha” and “Angry”), which often accompany or replace comments and may signal support or hostility in ways not captured by text alone. Future work could expand on this by integrating visual and textual data to explore multimodal patterns of stigmatizing engagement.

### Implications

The sentiment analysis across all topics revealed a generally negative sentiment in the comments. This uniform negativity underscores the hostile environment faced by researchers who study LGBTQIA+ populations and the LGBTQIA+ community on social media. The prevalence of negative sentiment and the nature of the themes identified in the comments have several implications for LGBTQIA+ researchers and the community.

First, as demonstrated in Topic 1, the most vitriolic and hostile reactions to LGBTQIA+ research virtual were often targeted directly at the researchers and LGBTQIA+ community. Navigating the predominantly negative sentiment and hostile language in these comments can have significant adverse effects on the well-being and productivity of researchers who study LGBTQIA+ populations. Therefore, it is important to recognize the potential mental health costs for researchers conducting such research, particularly those who are LGBTQIA+ themselves and often already face inequalities in scientific fields [18]. Retraumatization is a well-established risk for LGBTQIA+ researchers and clinicians, where engaging with discriminatory or harmful material in a research setting may trigger a traumatic response based on their own previous experiences [43]. Institutions can play a proactive role in mitigating these effects by offering formalized support such as training on managing virtual harassment, access to trauma-informed counseling services, and assistance with moderating public-facing content (eg, through communications or IT departments). Proactively preparing researchers—especially those working on marginalized topics—for potential hostility can help reduce psychological burden and prevent long-term disengagement.

Importantly, the early career authors in this paper reflected on the emotional labor and hesitation that followed these attacks, questioning whether continued public recruitment for LGBTQIA+-related studies was feasible or worth the emotional toll, and taking a break from recruitment to identify alternatives. Although we remain committed to this work, the intensity of the hostility has sparked conversations around modifying dissemination strategies or withdrawing from public engagement altogether, focusing instead on the use of private recruitment companies, which help minimize exposure to virtual hostility. Future research should examine how virtual hostility may influence researchers’ willingness to pursue similar topics over time, particularly as virtual visibility increases. We also recognize the privilege that we have had in being able to rely on private recruitment companies as an alternative, which may not be feasible for all researchers.

These risks may be especially consequential for early-career and junior scholars, who often lack institutional power and rely more heavily on external perceptions of professionalism, productivity, and reputational stability. Public attacks and stigmatizing commentary may jeopardize their willingness to remain in stigmatized research areas, particularly if they lack protective mentoring environments. Furthermore, many early-career researchers are building their public and professional identities virtually, where visibility is often necessary for networking and career advancement. The risk of reputational harm can be heightened during this career stage, reinforcing broader concerns about scientific workforce sustainability in marginalized research domains.

As previously mentioned, the vitriolic, anti-LGBTQIA+ language not only overwhelmed the comments sections but also spread to private messages sent to the research laboratory’s social media accounts. The inability to escape such derogatory terms and stigmatizing language, therefore, vastly increases the risk of traumatization when engaging in such work and thus could deter further research in this critical area [44]. The virtual hostility experienced by researchers both aligns with the MSC framework and evokes the SMC framework of stigmatization. In this case, even non-LGBTQIA+ researchers are regarded as part of the stigmatized group by proxy and treated with outrage due to their supposed role in undermining the legitimate social order. According to the SMC framework, this stigmatization of LGBTQIA+ and allied researchers alike could lead to a variety of responses ranging from outright avoiding similar projects to more full-heartedly committing to LGBTQIA+ research, based on their social and personal resources [27,49]. Therefore, it is vital for research organizations to provide increased institutional support and access to mental health resources for all researchers engaging in such work, but particularly for LGBTQIA+ researchers, so they may continue to use their valuable personal insights and passion to contribute to such vital research projects.

Second, Topic 2 demonstrates the tendency for comment sections related to LGBTQIA+ research to devolve into lengthy debates unrelated to the research topic itself. While slightly less negative than the other two topics, these Topic 2 comments were still highly antagonistic, with the cycle of escalating attacks between the two sides drowning out any genuine attempts to promote positivity or discussion. As this was the dominant response to these recruitment advertisements, it is quite evident that most of the engagement in these comment sections was fueled by this pattern of initial inflammatory statements followed by ongoing, vitriolic debate. In fact, several commenters even mentioned themselves that their debates may only be boosting the initial recruitment advertisement’s visibility and engagement.

Despite the predominantly negative sentiment across topics, it is essential to recognize the presence and potential impact of affirming responses. Even though affirming comments were quantitatively fewer and frequently embedded within adversarial exchanges, these comments represent important acts of solidarity, resistance, and social validation for LGBTQIA+ individuals and researchers. Prior research highlights that such visible affirmations can foster a sense of resilience and belonging among marginalized groups, potentially mitigating some of the psychological harms associated with stigma exposure [27,43]. Affirming responses may also serve as powerful social signals, helping to create safer virtual environments and encouraging further supportive interactions [25]. Therefore, future studies should specifically examine the qualitative effects of affirming virtual discourse, exploring how these interactions influence researchers’ emotional well-being, participants’ willingness to engage in research, and the broader community’s perceptions of LGBTQIA+ topics. Further, a temporal lens may be particularly valuable for identifying “trigger points” that amplify or mitigate virtual hostility toward LGBTQIA+ research. Future work should consider event-based or longitudinal data collection strategies to examine whether comment sentiment intensifies during key sociopolitical events (eg, policy debates and visibility campaigns) or shifts in response to broader cultural dynamics. Understanding these temporal dynamics may help researchers and institutions proactively prepare for periods of heightened hostility and foster more supportive virtual engagement.

However, the visibility of these debates likely did little to encourage greater participant engagement with the actual research project, based on both the content of the comments and prior research in this area. Previous research on news studies with similar comment sections indicated that the presence of such controversial debates may increase the post’s visibility, but does not really increase how many people actually engage with the posted study [28]. Therefore, it is reasonable to assume that such hostile debates feed partisans to comment sections but do not lead to greater numbers of potential participants engaging with the study. In fact, these hostile comment sections could possibly hinder participant recruitment efforts, as witnessing debates about one’s own identities likely does not encourage greater enthusiasm for participation among marginalized groups.

Through their socially viral yet acerbic nature, these stigmatizing comments place potential participants in the exact social predicaments postulated by the Social Management Communication Theory [27]. Unlike the researchers who have prior commitments to the project, these LGBTQIA+ social media users are introduced to the research within the context of personal stigmatization, which will frame their potential responses. Whether by posting vocally in the comments, participating to counter the virtual hostility, or avoiding the study entirely, potential participants exposed to these comments are likely to engage with the study as a reaction to the stigmatization rather than on their own terms. Therefore, to avoid further stifling or distorting research on this already underrepresented community, researchers should consider how current policies on different platforms may impact LGBTQIA+ recruitment and also advocate for the adoption of future-looking policies that reduce these hostile debates on recruitment advertisements.

Third, the comments in Topic 3 demonstrate how these responses to virtual LGBTQIA+ are inseparably linked to their context within the larger social-political debates and divides in a society. While comment sections near universally trend toward negative sentiment, the degree of negativity is often determined both by how the technological structures of the platform drive engagement and by how the commenters’ political environment frames the debate [7]. From this perspective, many of the Topic 3 comments reflect not only an attempt to attack the LGBTQIA+ community but also a desire to signal to perceived peers that they are on the “correct” side of a sociopolitical divide in the United States that spans many issues and topics [42].

While highly visible names and identity markers on Facebook likely contribute to this specific dynamic, anonymity would likely not solve this problem either, as anonymous comment sections simply experience different incentives toward negativity [50]. The deeper issue in these cases is that social media platforms consistently heighten negativity and polarization toward culturally controversial topics. Reflecting the Social Identity Theory, the changing cultural dynamics in many parts of the world, from Brazil to East Asia, have positioned debates regarding the very validity of LGBTQIA+ identities and rights as central to these political controversies [22,51]. Given this challenging environment toward LGBTQIA+ topics, only further highlighted by our findings here, there is an evident pressing need for research teams and institutions to recognize the demands placed upon LGBTQIA+ researchers, to develop strategies to mitigate negative sentiments, and to foster a more supportive and respectful discourse.

Further, while our analyses did not directly examine intersectionality, we acknowledge that hostility toward LGBTQIA+ research may vary based on intersections with other marginalized identities such as race, ethnicity, religion, and immigration status. Due to the anonymous and limited nature of commenter profiles, we were unable to assess how such intersecting identities influenced either the content or targets of hostile comments. Similarly, while the researchers’ identities were not explicitly mentioned in most comments, future research should explore how perceived or actual intersections of researcher identity—such as being LGBTQIA+, a person of color, or an immigrant—may shape the nature and intensity of virtual hostility. Notably, the principal investigators leading this research are themselves immigrants and persons of color, highlighting the importance of recognizing how researchers’ lived experiences may intersect with the content and reception of their work. Intersectionality theory [52-54] provides a critical lens for understanding how multiple systems of oppression interact, and integrating this perspective into future virtual stigma research may reveal important nuances in public responses and inform more inclusive strategies for researcher protection and participant engagement.

Although our analysis focused exclusively on Facebook comments, we acknowledge that platform architecture, moderation policies, and user demographics can significantly shape the tone, content, and volume of virtual hostility. For instance, Facebook’s real-name policy may facilitate identity signaling through overt moral outrage or attempts to affirm in-group belonging, while platforms like Reddit or X (formerly Twitter)—where anonymity or pseudonymity is more common—may foster different forms of incivility, such as trolling or dog-whistle language. Moreover, Facebook’s algorithmic curation and comment threading may amplify polarizing debates, as evidenced by the prevalence of adversarial exchanges (Topic 2) in our dataset.

Future research should systematically compare hostility patterns across platforms to assess whether LGBTQIA+-focused research is uniquely targeted or if similar dynamics emerge across other stigmatized health domains. Cross-platform analyses would allow researchers to evaluate how features like anonymity, character limits, moderation stringency, or community norms influence stigma communication. Such insights could inform tailored strategies for protecting LGBTQIA+ researchers and participants—ranging from platform-specific recruitment policies to advocacy for improved moderation tools. Understanding these affordances is essential for developing responsive institutional guidelines and equipping researchers with evidence-based recommendations for safer virtual public engagement.

### Recommendations

The overwhelming negativity across all three of these topics confirms previous findings regarding virtual hostility directed at researchers and thus suggests that there is a dire need to adopt guidelines and policies to deal with virtual hostility, particularly for researchers working on minoritized or politically contested topics [11,14]. Institutional responses to such virtual hostility have been reported to be inadequate, so our team considered deeply the different roles research institutions, individual teams, policy makers, and the broader public could play in creating a healthier environment for LGBTQIA+ research and the LGBTQIA+ community more broadly [55]. Based on these findings, we make four primary recommendations for a more constructive path forward: enhanced institutional protection, tailored support for early-career scholars, proactive policy advocacy, and broader public awareness efforts.

First, to support LGBTQIA+ researchers and their work, it is essential to develop robust institutional support systems that offer comprehensive psychological assistance and training on navigating the ethical and legal complexities of LGBTQIA+ research. This includes offering workshops or embedded training modules for faculty, students, and staff on identifying and responding to virtual hostility, trauma-informed peer support, and reporting or moderating harmful content. Institutions should also ensure that social media or communications offices are equipped to assist researchers facing digital harassment, so that individual scholars do not bear the full burden of managing public backlash.

We specifically recommend that educational and research institutions provide preparatory training on virtual harassment and increase access to third-party recruitment organizations when studies involve potentially sensitive or stigmatized topics. Several members of our team reported experiencing heightened emotional strain due to the unexpected and targeted nature of the virtual hostility documented in this project. Providing training in advance can help mitigate this vulnerability by equipping researchers with a clearer understanding of virtual hostility, strategies for emotional preparedness, and protective measures. Institutions can also allocate resources for the use of third-party recruitment services, which serve as a protective buffer by minimizing direct contact with adversarial actors and reducing the likelihood of personal information being exposed.

Second, institutions must also recognize and address the unique vulnerabilities of early-career researchers. For graduate students, postdoctoral researchers, and untenured faculty conducting LGBTQIA+-focused work, virtual hostility may carry disproportionate career consequences—discouraging them from pursuing socially impactful but politically fraught research questions. Universities and research organizations should therefore implement protective mentorship structures, prioritize inclusion of such scholars in resilience-building networks, and offer concrete incentives (eg, release time, bridge funding, or public support) to retain talent in LGBTQIA+ research areas. Without these safeguards, the emotional toll and reputational risks may drive early-career scholars away from equity-oriented work. Professional societies and academic consortia should also advocate for the mechanisms of the National Institutes of Health and the National Science Foundation to explicitly address the costs—emotional, reputational, and logistical—of conducting research on politically charged or marginalized populations.

Third, research institutions, teams, and individuals should advocate for improved private and public policies to increase protection for LGBTQIA+ communities, both virtual and offline. As previously mentioned, research institutions should advocate that social media companies drastically improve content moderation policies to encourage constructive engagement with research advertisements, protect researchers from virtual hostility, and limit the spread of hate speech and misinformation. We specifically advocate for enhanced content moderation by social media companies in the comment sections of research-related posts, particularly in response to hate speech targeting the LGBTQIA+ community. Given the recent trend of reduced content moderation across platforms, we recommend, at a minimum, that platforms allow researchers to disable comments on recruitment posts and advertisements (a feature already available on some sites). This simple but impactful step could help limit researcher harassment and protect potential participants from exposure to hate speech. In the absence of such features, we encourage researchers to consider recruiting only on platforms that offer either comment disabling or sufficient moderation, both to safeguard their studies and to signal the importance of institutional accountability in digital spaces.

Finally, raising awareness about the challenges faced by LGBTQIA+ researchers and highlighting their contributions can inspire others to similarly engage in such impactful research efforts. This includes university-led media campaigns, internal recognition programs, and community engagement initiatives that elevate the visibility and value of equity-driven research. Engaging the public through transparent, compassionate messaging may also counter prevailing misinformation and reduce stigma around LGBTQIA+ topics and the researchers studying them. Ultimately, as this project and broader scholarship on social identity and stigma show, social media platforms will continue to serve as sites of virtual hostility toward LGBTQIA+ individuals so long as LGBTQIA+ identities remain politically contested and structurally marginalized. To meaningfully reduce harassment targeting researchers and LGBTQIA+ communities alike, efforts must extend beyond platform-level moderation to address the systemic sociopolitical conditions that fuel hostility, and that are amplified by the engagement-maximizing algorithms that dominate digital spaces.

### Conclusions

This study provides an overview of the thematic content and sentiment of comments directed at LGBTQIA+ researchers and recruitment efforts. By documenting consistent negative sentiment signaling social identity through the stigmatization of LGBTQIA+ individuals and researchers in virtual spaces, our findings reinforce existing theoretical models on stigma within an underresearched population and medium. As these frameworks suggest, such stigmatization can negatively affect mental health and dissuade future participation. Our findings highlight the need for targeted structural interventions to support researchers and promote a more inclusive and respectful dialogue around LGBTQIA+ issues. Future research should explore the long-term impacts of such hostile comments on researchers and participants, as well as the effectiveness of various strategies in mitigating these negative sentiments. Additionally, the affirming responses documented in this study, despite being fewer in number, indicate potential avenues for fostering supportive engagement and reducing stigma. Further qualitative research on these affirming interactions could illuminate effective strategies to enhance resilience and well-being among LGBTQIA+ researchers and community members. Future research should also explore interventions to mitigate the negative impact of such comments and promote a supportive atmosphere for LGBTQIA+ research. By raising awareness of and addressing these challenges, we can work toward a more supportive environment for LGBTQIA+ research and advocacy.

## Abbreviations

LDA: latent Dirichlet allocation
LGBTQIA+: lesbian, gay, bisexual, transgender, queer/questioning, intersex, asexual
LIWC: Linguistic Inquiry and Word Count
MSC: Model of Stigma Communication
SMC: Stigma Management Communication Theory

## Data Availability

The datasets generated or analyzed during this study are not publicly available due to concerns about the safety, privacy, and well-being of affected individuals, including researchers, participants, and commenters, but are available from the corresponding author on reasonable request.
